# Oral Delivery of miR146a Conjugated to Cerium Oxide Nanoparticles Improves an Established T Cell-Mediated Experimental Colitis in Mice

**DOI:** 10.3390/pharmaceutics16121573

**Published:** 2024-12-09

**Authors:** Anisha Apte, Pujarini Dutta Dey, Srisaianirudh Reddy Julakanti, Monica Midura-Kiela, Stacy M. Skopp, Jimena Canchis, Tobias Fauser, James Bardill, Sudipta Seal, David M. Jackson, Fayez K. Ghishan, Pawel R. Kiela, Carlos Zgheib, Kenneth W. Liechty

**Affiliations:** 1 Laboratory for Fetal and Regenerative Biology, Department of Surgery, University of Arizona Tucson College of Medicine, Banner Children’s at Diamond Children’s Medical Center, 1656 E Mabel St, Rm 230, Tucson, AZ 85721, USA; 2 Department of Pediatrics, Daniel Cracchiolo Institute for Pediatric Autoimmune Disease Research, Steele Children’s Research Center, University of Arizona Health Sciences Center, Tucson, AZ 85621, USA; 3 Laboratory for Fetal and Regenerative Biology, University of Colorado School of Medicine and Children’s Hospital Colorado, Aurora, CO 80045, USA; 4 Advanced Materials Processing and Analysis Center, Nanoscience Technology Center, University of Central Florida, Orlando, FL 32826, USA; 5 Ceria Therapeutics, Inc., Tucson, AZ 85721, USA; 6 Department of Immunobiology, University of Arizona Health Sciences Center, Tucson, AZ 85621, USA

**Keywords:** inflammatory bowel disease, microRNA, nanomedicine, oxidative stress

## Abstract

**Background:** Dysregulated inflammation and oxidative stress are strongly implicated in the pathogenesis of inflammatory bowel disease. We have developed a novel therapeutic that targets inflammation and oxidative stress. It is comprised of microRNA-146a (miR146a)-loaded cerium oxide nanoparticles (CNPs) (CNP-miR146a). We hypothesized that oral delivery of CNP-miR146a would reduce colonic inflammation in a mouse model of established, chronic, T cell-mediated colitis. **Methods:** The stability of CNP-miR146a and mucosal delivery was assessed in vitro with simulated gastrointestinal fluid and in vivo after oral gavage by quantitative real-time RT-PCR. The efficacy of orally administered CNP-miR146a was tested in mice with established colitis using the model of adoptive naïve T-cell transfer in recombinant activating gene 2 knockout (Rag2^−/−^) mice. Measured outcomes included histopathology; CD45^+^ immune cell infiltration; oxidative DNA damage (tissue 8-hydroxy-2′-deoxyguanosine; 8-OHdG); expression of IL-6 and TNF mRNA and protein, and flow cytometry analysis of lamina propria Th1 and Th17 cell populations. **Results:** miR146a expression remained stable in simulated gastric and intestinal conditions. miR146a expression increased in the intestines of mice six hours following oral gavage of CNP-miR146a. Oral delivery of CNP-miR146a in mice with colitis was associated with reduced inflammation and oxidative stress in the proximal and distal colons as evidenced by histopathology scoring, reduced immune cell infiltration, reduced IL-6 and TNF expression, and decreased populations of CD4^+^Tbet^+^IFNg+ Th1, CD4^+^RorgT^+^IL17^+^ Th17, as well as pathogenic double positive IFNg^+^IL17^+^ T cells. **Conclusions**: CNP-miR146a represents a novel orally available therapeutic with high potential to advance into clinical trials.

## 1. Introduction

Inflammatory bowel disease (IBD) is an immune-mediated chronic disease of the gastrointestinal system that affects more than five million people worldwide [1]. Despite recent advances in medical and surgical therapies, IBD remains associated with a high economic burden [2]. Biologic agents targeting tumor necrosis factor (TNF), integrins, or interleukin-23 (IL-23) have revolutionized the treatment of IBD. However, their use is costly and is associated with an increased risk of infections, allergic reactions, injection site reactions, autoimmune disorders, increased risk of some cancers, heart failure, and neurological and hepatic adverse effects, and their efficacy may wane due to immunogenicity and antibody development [3]. Additionally, approximately 30% of IBD patients fail to respond to these biologics altogether and are left with few alternatives [4]. While the emerging orally-available Janus kinase (JAK) inhibitors, which attenuate multiple cytokine signaling pathways, represent an effective therapy for many patients with moderate to severe IBD, their use is also not without adverse effects or limitations, especially in patients over 65 years of age, those with increased risk of major cardiovascular problems, long-time smokers, or those with increased risk of cancer [5]. Thus, there is a pressing need for additional novel therapeutics with distinct mechanisms of action to effectively treat IBD.

The pathophysiology of IBD is complex and involves genetic susceptibility, environmental exposures, immune dysfunction, and the gut microbiome. The genetic component of the development of IBD involves over 200 identified genetic loci, of which 80–90% are localized within non-coding regions and are believed to exert their pathogenic effects through modulation of gene expression [6,7]. Moreover, dysregulation of microRNAs (miRNAs) and long non-coding RNAs (lncRNAs), the two main classes of non-coding RNAs, is also believed to represent a major contributor to the uncontrolled inflammatory response in IBD [8]. miRNAs are small RNAs that play regulatory roles in transcriptional and post-transcriptional gene regulation. Recent studies have demonstrated abnormal expression of several miRNAs in IBD, many of which are involved in the regulation of molecular pathways associated with immune responses, including the nuclear factor kappa-light-chain-enhancer of activated B cells (NF-kB) pathway [8,9].

One of these miRNAs is miR146, which is encoded in humans by the *MIR-146A* gene and inhibits nuclear translocation of NF-kB by targeting the protein’s interleukin-1 receptor = associated kinase 1 (IRAK1) and tumor necrosis factor receptor-associated factor 6 (TRAF6) within the signaling pathway of cell surface toll-like receptors (TLRs). miR146a suppresses the expression of inflammatory genes and is essential to mediating and resolving inflammatory damage [10,11,12]. A recent small study showed that *rs2910164* polymorphism in *MiR-146a* genes was significantly associated with increased risk and severity of UC [13]. Interestingly, there are few animal studies that link miR146a expression with increased inflammation and susceptibility to inflammatory colitis [14,15]. This includes a study demonstrating that mice with miR146a deficiency were less susceptible to chemical-induced colitis [14]. A more recent study, utilizing the same model, demonstrated the opposite effect, in which miR146a deficient mice exhibited a more severe colitis [16]. More recently, there have been an abundance of data demonstrating that miR146a attenuates experimental colitis and inflammation-associated colon cancer [17,18,19,20].

A further layer of complication is added by the fact that utilizing miRNAs as orally bioactive therapeutics is challenging given the unstable nature of miRNAs and their susceptibility to degradation. We have developed a novel method for stabilizing and delivering miRNA by conjugating it to a cerium oxide nanoparticle (CNP). miRNAs are chemically conjugated to CNPs at the 3′ end, with the use of an amide linker through 1,1′-carbonyldiimidazole (CDI) chemistry. This leaves the 5′ end of the miRNA available to bind to target mRNA. Theoretically, chemical conjugation with CNP and charge composition allows the miRNA to directly enter the cell cytoplasm through endosomes and escape lysosomal degradation [21].

In addition to stabilizing the miRNA, CNP provides synergistic effects by scavenging free radicals and reducing reactive oxidative species (ROS) [22]. CNPs, due to their physical and chemical redox properties, have the ability to mimic the activity of antioxidative enzymes, like catalase or superoxide dismutase, to neutralize ROS and improve redox balance in cells [23,24,25]. There are two forms of cerium oxide—ceric oxide CeO_2_ (Ce^4+^) and cerous oxide Ce_2_O_3_ (Ce^3+^). The antioxidant activities of cerium oxide depend on the Ce^3+^/Ce^4+^ ratio at the particle surface, with high surface Ce^3+^ exhibiting superoxide dismutase-mimetic activity and high surface Ce^4+^ displaying greater catalase activity. On a nanoscale, Ce^3+^ atoms occupy the majority of oxygen vacancies at the surface due to charge compensation, which provides CNP greater antioxidant properties. In prior studies, we have found that the shape of CNP influences Ce^3+^ surface percentage, as well as miRNA loading capacity, however all formulations demonstrated negligible cytotoxicity. Multiple toxicity studies have demonstrated the biocompatibility and safety of CNPs [26,27,28,29]. CNP-miR146a conjugates additionally demonstrated greater chemical stability and persistent ROS scavenging ability when compared to miR146a conjugated to other nanoparticles, including gold and silica [21].

We have previously demonstrated that the CNP-miR146a conjugate was effective in significantly reducing inflammation in animal models of acute lung injury and diabetic wounds in intradermal, topical, and intratracheal applications [30,31]. We have recently demonstrated the efficacy of intrarectal CNP-miR146a in 2,4,6-Trinitrobenzenesulfonic acid (TNBS)-induced acute colitis [32]. However, preclinical efficacy of orally administered miR146a in the established phase of chronic progressive T cell-mediated colitis, in an experimental setting resembling a clinical scenario, has not yet been established. Thus, this study was designed to test the effects of orally-delivered CNP-miR146a in Rag2^−/−^ mice with adoptive CD4^+^CD45RB^HI^ T cell transfer colitis, a model developed by Powrie et al. [33], which requires both spontaneous proliferation of T cells driven by microbiota-derived innate signals and antigen-specific T cell proliferation [34].

We demonstrate CNP-miR146a stability in simulated gastric and intestinal fluid, which indicates that CNP-miR146a can survive the pH changes of the gastrointestinal system and show efficient delivery of miR146a to the intestinal mucosa. In vivo, therapeutic administration of CNP-miR146a in mice with established chronic colitis induced by adoptive T-cell transfer in Rag2^−/−^ mice significantly improved histological and immunological symptoms of the disease. These data collectively suggest that miR146a conjugated to CNP represents an orally available viable alternative or adjunct treatment of IBD.

## 2. Materials and Methods

### 2.1. Synthesis of CNP-miR146a

CNP-miR146a was synthesized as previously described [22]. In brief, CNPs were synthesized via chemical hydrolysis then conjugated to miR146a using 1′-1′ carbonyldiimidazole (CDI) chemistry to covalently bind the miR146a amino group to the CNP hydroxyl group. CNP-miR146a concentration was assessed by Quant-iT^TM^ (ThermoFisher Quant-iT™ microRNA Assay Kit, Waltham, MA, USA) to determine miR146a concentration and by inductively coupled plasma mass spectrometry (ICP-MS) to determine cerium concentration. The conjugated CNP-miR146a was diluted in sterile phosphate buffer saline (PBS) to a concentration of 0.025 ng/mL and stored at −20 °C.

### 2.2. CNP-miR146a Stability in Simulated Gastrointestinal Fluid

This study was designed after a model proposed by an international consensus on performing standardized static in vitro digestion studies [35]. Simulated gastric fluid (SGF) and simulated intestinal fluid (SIF) stock solutions were prepared to mimic in vivo physiological salt concentrations and filter-sterilized (Table 1).

The gastric condition was created by mixing 2.5 mL PBS containing 950 ng of CNP-miR146a in a 1:1 ratio with a preparation of SGF combined with HCl to a final pH of 1.5. This mixture was placed on a shaker at 200 rpm at room temperature for 2 h to mimic the average transit time in the stomach. Aliquots of 40 µL were withdrawn at the following time points: 0 h, 0.5 h, 1 h, 1.5 h, 2 h. These 40 µL aliquots were neutralized to pH 7 with 5 µL of 1M NaHCO_3_. At 2 h, the starting solution was mixed in a 1:1 ratio with a preparation of SIF, bovine trypsin (Sigma, St Louis, MO, USA, EC 3.4.21.4), and NaOH (Sigma, St Louis, MO, USA, CAS#1310-73-2) combined to create a final pH of 7, replicating transition to the intestine. The addition of trypsin was intended to mimic the proteolytic activity of enzymes in the small intestine, of which trypsin is the most abundant. An amount of 1 mg (8300 BAEE units) of bovine trypsin was reconstituted in 1 mL of RNAse-free water, of which 60 µL were used to achieve a final enzyme activity concentration of 100 BAEE units/mL. This mixture was replaced on the shaker at 200 rpm at room temperature for four additional hours to mimic the average transit time in the intestine and colon. Aliquots of 40 µL were withdrawn at the following additional time points: 2 h, 3 h, 4 h, 5 h, 6 h. These 40 µL aliquots were neutralized with 4 µL of 10× protease inhibitor (Pierce^TM^, Waltham, MA, USA, REF A32965) to inhibit continued trypsin activity. This experiment was repeated three times separately. All aliquots were immediately snap-frozen in liquid nitrogen upon collection and stored in a −80 °C freezer until ready for analysis. Quantitative real-time polymerase chain reaction (qRT-PCR) was used to detect miR146a in a 1:10 dilution of each aliquot cDNA.

### 2.3. In Vivo Verification of CNP-miR146a Delivery After Oral Administration

All animal studies were approved by the Institutional Animal Care and Use Committee (IACUC, protocols 07-126 and 2022-0923) at the University of Arizona College of Medicine. Animal care was performed by trained veterinarians and technologists according to the NIH Guide for the Care and Use of Laboratory Animals. Male C57BL/6J (Jackson Laboratory) mice of 7–8 weeks of age mice underwent oral gavage of 5 ng of CNP-miR146a in 200 mL PBS or 200 mL PBS alone. Mice were euthanized at 4, 6, or 24 h following oral gavage to cover the complete murine gastrointestinal transit time [36]. Terminal ileum (1 cm before cecum), cecum, and proximal and distal colon were dissected, flushed with PBS to remove enteric contents, and opened longitudinally. Tissue samples were snap-frozen in liquid nitrogen and stored in a −80 °C freezer until ready for RNA extraction and quantitative miR146a analysis by qRT-PCR.

### 2.4. Adoptive T-Cell Transfer Colitis

Adoptive transfer of naïve CD4^+^CD45RB^HI^ T-cells was carried out as previously described and performed [37]. Male and female eight- to ten-week-old immunodeficient Rag2^−/−^ mice on a colitis-susceptible 129/SvEV background (originally from Taconic) were randomly assigned to receive an intraperitoneal (IP) injection of either prepared donor T-cells or PBS. To prepare donor T-cells, spleens from wild-type (WT) 129/SvEV mice were collected into complete medium (RPMI + GlutaMAX, Gibco, Grand Island, NY, USA) containing 10% fetal bovine serum (FBS, HyClone, Logan, UT, USA), 5% penicillin streptomycin (HyClone), and 5% non-essential amino acids (NEAA, HyClone). Splenocytes were released from tissue through mechanical mashing using the blunt end of a 5 mL syringe plunger and the prepared cell suspension was passed through a 70 mm cell strainer (Alkali Scientific, Inc., Fort Lauderdale, FL, USA). Following red cell lysis (Pharm Lyse, BD, Franklin Lakes, NJ, USA), released splenocytes were enriched for CD4^+^ population using negative selection kit (Stem Cell EasySep™ Mouse CD4+ T Cell Isolation Kit Cat#19752; EasyEights™ EasySep™ Magnet (catalog#18103)) followed by labeling with rat anti-mouse CD4-PE and rat anti-mouse CD45RB-FITC antibodies (BD, cat#’s 553059 and 553100, respectively), and flow sorting of the top 40% of CD45RB-expressing cells (CD4^+^CD45RB^Hi^) using the FACSAria III (BD Biosciences, San Jose, CA, USA). Mice were injected IP with 0.5 × 10^5^ naïve CD4^+^CD45RB^Hi^ T cells in 100 mL of sterile PBS. Control mice received PBS alone. Once a week, mice were monitored for symptoms of disease and from 6 to 10 weeks following T-cell transfer, fecal pellets were collected to measure fecal lipocalin-2 concentration. Fecal lipocalin-2 is a marker of intestinal inflammation that has previously been demonstrated to be elevated for patients with active Crohn’s disease [38] and has been validated as a marker for identifying mice that have developed colitis in this model [39,40]. Mice with fecal lipocalin-2 levels ≥ 900 ng/g were determined to have developed colitis and were assigned to receive daily oral gavage of either 5 ng of CNP-miR146a in 200 mL of sterile PBS or 200 mL of sterile PBS alone for five days. Sham Rag2^−/−^ mice that received IP PBS were administered 200 mL of oral PBS for the same time period to serve as controls. A set of age-matched 129/SvEV mice were euthanized as an additional untreated immunocompetent naïve wild-type (WT) control. The following cohorts were used in this study: naïve WT (no intervention), sham (Rag2^−/−^; PBS IP + PBS gavage), colitis + PBS (Rag2^−/−^ + T cells + PBS gavage), and colitis + CNP-miR146a (Rag2^−/−^ + T cells + CNP-miR146a gavage). Mice were euthanized 24 h following the fifth oral administration. Colons were dissected from cecum to the anal verge and separated into proximal and distal segments. Tissues were flushed with PBS to remove enteric contents and processed either for RNA and protein extraction, histology, or lamina propria immune cell isolation.

### 2.5. Fecal Lipocalin-2 Evaluation

Fecal pellets collected from Rag2^−/−^ mice were snap-frozen in liquid nitrogen and stored at −80 °C until ready for use. Fecal suspensions were prepared from fecal samples by recording the weight of each sample, suspending the sample in 1 mL PBS containing 0.1% Tween 20, and mixing the sample on a shaker at 900 rpm for 30 min until fully resuspended. Then, centrifugation was carried out on the sample at 12,000 rpm at 4 °C for 10 min. Collected supernatants were diluted with 1× Reagent Diluent 2 at 1:500 ratio and used to detect lipocalin-2 with enzyme-linked immunosorbent assays (ELISA) kit according to the manufacturer’s instructions (R&D Systems™ Mouse Lipocalin-2/NGAL DuoSet ELISA kit, Minneapolis, MN, USA). Quantification of protein was detected by measuring absorbance at 450 nm with SpectraMax plate reader (Molecular Devices, San Jose, CA, USA).

### 2.6. Evaluation of Colonic Histology

Colons were rolled into “Swiss rolls” and fixed in a histology cassette in 10% formaldehyde for 24–48 h at room temperature before dehydrating in a 70% ethyl alcohol and storing at 4 °C. Dehydrated tissues were embedded in paraffin blocks and sectioned at 4 mm. Mounted sections were deparaffinized and stained with hematoxylin and eosin (H&E). Tissue sections were viewed and photographed at 20× and 100× using brightfield microscopy with the digital Keyence BZ-X microscope and scored with a scoring system previously designed for the adoptive T cell transfer colitis model [41]. Briefly, an injury score of 1–5 was assigned based on the severity and extent of inflammatory cell infiltration in addition to the degree of epithelial changes and architectural distortion (Table 2).

### 2.7. Immunohistochemical Analysis of CD45^+^ Immune Cell Infiltration

Inflammatory cell infiltration of colon tissue was evaluated by CD45^+^ staining performed by the Tissue Acquisition and Cellular/Molecular Analysis Histology Lab at the University of Arizona. Unstained slides sectioned at 4 µm were deparaffinized and incubated with anti-CD45 primary antibody (BD Biosciences, Cat#550539, 1:100 dilution, Franklin Lakes, NJ, USA) overnight followed by secondary antibody and chromogen staining. Stained tissue sections were viewed and ten random images at 200× were photographed from the entire tissue specimen using brightfield microscopy with the digital Keyence BZ-X microscope (Itasca, IL, USA). Percent tissue area of CD45^+^ staining was quantified using BZ-X800 imaging software (Osaka, Japan).

### 2.8. Immunofluorescent Analysis of 8-Hydroxy-2′-Deoxyguanosine (8-OHdG) as a Marker of Oxidative Stress

Oxidative stress within colonic tissue was evaluated by measuring 8-hydroxy-2′-deoxyguanosine (8-OHdG) in a subset of Rag2^−/−^ colitic mice treated with PBS and CNP-miR146a. Unstained tissue slides sectioned at 4 µm were deparaffinized and incubated in xylene for ten minutes. This process was repeated for a total of three times, and then sections were rehydrated through a graded ethanol series (100%, 95%, 90%, 80%, and 70%) for five minutes each and cleaned with distilled water. An amount of 100 µg/mL RNase A (Sigma) was added to tissue sections and incubated for one hour at 37 °C in TEN buffer (10mM Tris-HCl (pH 7.4) and 1 mM ethylenediaminetetraacetic acid ((EDTA) pH 7.6, 400 mM NaCl)). Following this, sections were rinsed with distilled water and then incubated with 10 μg/mL proteinase K (Boehringer, Inc., Indianapolis, IN, USA) dissolved in 100 mM Tris-HCl (pH 8.0) and 5 0mM EDTA (pH 7.6) for ten minutes at room temperature. Slides were rinsed with distilled water and then incubated in 2M HCl for five minutes to denature DNA before neutralizing them with 1M Tris-base for an additional seven minutes. Slides were washed with PBS and then blocked with a mouse-on-mouse blocking solution (Vector Labs, Newark, CA, USA). Tissue sections were incubated with a primary monoclonal antibody specific for 8-OHdG (e.g., anti-8-OHdG antibody, Santacruz Biotechnology sc-66036, Dallas, TX, USA, (15A3) diluted 1:100 in PBS) overnight at 4 °C. After rinsing with PBS, sections were incubated with a goat anti-Mouse IgG conjugated to Alexa Fluor Plus-594 (Invitrogen, Waltham, MA, USA, A32742, diluted 1:400) for 30 min at room temperature. Sections were washed with PBS, counterstained with DAPI, and mounted in ProLong Gold mounting medium (Invitrogen). Images for quantitative analysis were captured using epifluorescence microscope (EVOS M700, Invitrogen) and a confocal microscope for a capture of representative images (Olympus FluoView FV10i). Nine to ten random images at 20× were captured from each entire tissue specimen, and the intensity of 8-OHdG staining was quantified for mean fluorescent intensity (MFI) using ImageJ software v1.54.

### 2.9. Colonic mRNA and miRNA Expression Analysis

Total RNA was extracted from frozen intestinal tissue using Qiazol (Qiagen, Germanton, MD, USA) with mechanical homogenization according to the manufacturer’s instructions. Total RNA was quantified using a Synergy LX multi-model plate reader (BioTek, Houston, TX, USA) and treated with DNAse (Invitrogen, ThermoFisher, Waltham, MA, USA) prior to reverse transcription reaction and cDNA synthesis with High-Capacity cDNA Reverse Transcription Kit (Applied Biosystems, ThermoFisher, Waltham, MA, USA). qRT-PCR was performed with CFX96 Touch Real-Time PCR Detection System (BioRad, Hercules, USA) using PrimeTime Gene Expression Master Mix (Integrated DNA Technologies, Coralville, IA, USA) to evaluate relative gene expression of interleukin-6 (IL-6) (ThermoFisher Mm00446190_m1 IL6), tumor necrosis factor (TNF) (ThermoFisher Mm00443258_m1 Tnf), and matrix metalloproteinase 9 (MMP-9) (ThermoFisher Mm00442991_m1 mmp9), using glyceraldehyde 3-phosphate dehydrogenase (GAPDH) (ThermoFisher Mm99999915_g1 gapdh) as an endogenous control. For evaluation of miRNA, RNA was diluted to 5 ng/mL and converted to miR146a cDNA (ThermoFisher RT&TM snRNA 000468) using TaqMan microRNA reverse transcriptase kit (Applied Biosystems, ThermoFisher) and amplified using the qRT-PCR steps described above. U6 cDNA was used as an endogenous control (ThermoFisher RT&TM snRNA 001973).

### 2.10. Colonic Proinflammatory Cytokine Protein Analysis

Frozen colon tissue samples were homogenized in radioimmunoprecipitation assay (RIPA) lysis buffer (RIPA Lysis and Extraction Buffer; ThermoFisher Cat# 89900) and protease inhibitors. Samples were centrifuged, and the collected supernatant was used for total protein concentration using a Pierce BCA assay (ThermoFisher Cat# A53225) and for IL-6 and TNF protein analysis using ELISA (R&D Systems™ Mouse IL6 DuoSet ELISA kit and Mouse TNFa DuoSet ELISA kit) with standardized loading of 200 mg total protein for TNF detection and 400 mg total protein for IL-6 detection.

### 2.11. Isolation of Colonic Lamina Propria Immune Cells and Flow Cytometry Analysis

Dissected colons were gently flushed with Ca^2+^/Mg^2+^-free Hank’s balanced salt solution (HBSS; Gibco), opened longitudinally, cut into 1 cm segments, and further rinsed with HBSS. Tissue fragments were then incubated at 37 °C with shaking for 20 min followed by vigorous vortexing for 1 min in HBSS with 10mM HEPES and 2% FBS (HHF) supplemented with 2mM EDTA. Resulting fragments were digested for 20 min at 37 °C and shaken using 100 U/mL type 1 collagenase (Worthington Biochemical Corporation, Lakewood, NJ, USA) in HHF containing 40 μg/mL DNase I (Gold Biotechnology, Olivette, MO, USA). The tissue pieces were then removed and vigorously vortexed for 1 min. The released cells were filtered through a 100 μm filter (BD Falcon) and collected into full RPMI 1640 media supplemented with 10% FBS, 100 μg/mL streptomycin, 100 μg/mL penicillin, and 2 mM L-glutamine (all from Gibco/Invitrogen). Trypan blue exclusion and a hemocytometer were used to count viable cells, and cell concertation was adjusted with 2% FBS, 0.01% sodium azide PBS (FACS buffer) to stain for flow cytometry. The cells were blocked using an antibody (Mouse BD Fc Block™ 553141). Antibodies against cell surface markers were incubated for 30 min at 4 °C in the dark. For IL-17, IFNg, and T-bet staining, the cells from colonic lamina propria were fixed and permeabilized on ice with Fixation and Permeabilization Buffer following manufacturer’s protocol (eBioscience™, San Diego, CA, USA, Fixation/Permeabilization Concentrate Catalog number: 00-5123-43 eBioscience™; Fixation/Permeabilization Diluent Catalog number: 00-5223-56; Permeabilization Buffer 10X 00-8333-56). Cells were washed three times with cold PBS and incubated with anti-IL17-FITC (Biolegend 506907), anti-RORgT-BV421(BD 562894), anti-mouse IFNγ-PE/Dazzle™ 594 (Biolegend 505845), and anti-Tbet-APC (Biolegend 6448130) antibodies for 30 min on ice in the dark. The cells were then washed two times and analyzed by flow cytometry. All data were acquired using the LSR Fortessa flow cytometer (Becton Dickinson, Franklin Lakes, NJ, USA), and data were analyzed using FlowJo, https://www.flowjo.com/.

## 3. Statistical Analyses

All statistical analyses were performed in GraphPad Prism 9.5.1 (La Jolla, CA, USA). The Grubbs method with an alpha value set to 0.05 was used to identify and remove outliers. An unpaired Student’s *t*-test was used to compare two cohorts, while a one-way ANOVA test followed by a post hoc Tukey’s test was used to compare multiple cohorts. Histology scores were compared with the Kruskal–Wallis Test. The alpha value < 0.05 was considered statistically significant.

## 4. Results

### 4.1. CNP Formulation of miR146a Is Stable in Simulated Gastrointestinal Fluid

**Figure 1:**
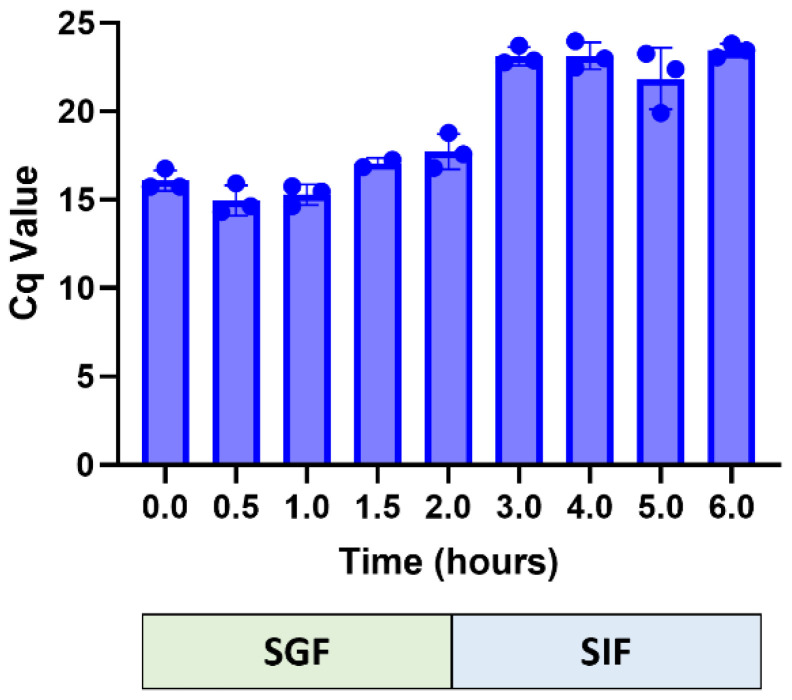
Stability of miR146a in CNP-miR146a in simulated gastrointestinal fluid. CNP-miR146a was added to simulated gastric fluid (SGF), which after 2 h of incubation, was adjusted to represent small intestinal environment (simulated intestinal fluid, SIF). Aliquots were removed at indicated times and used for qRT-PCR analysis of miR146a.

miR146a was detected by qRT-PCR of 1:10 cDNA made from aliquots of SGF and SIF obtained from every time interval of the study, including the sample collected immediately upon CNP-miR-146a addition (at hour 0) and at the completion of the study (6 h). miR146a could be detected in both samples and remained stable in acidic conditions of pH 1.2 (SGF). Immediately following the transition to SIF (pH 7), Ct values increased, which indicated a degree of initial miR146a degradation/hydrolysis upon addition of NaOH, but the miR146a abundance remained stable thereafter (Figure 1).

### 4.2. Mucosal miR146a Transiently Increases in the Mouse Ileum and Colon After Oral Administration of CNP-miR146a

**Figure 2:**
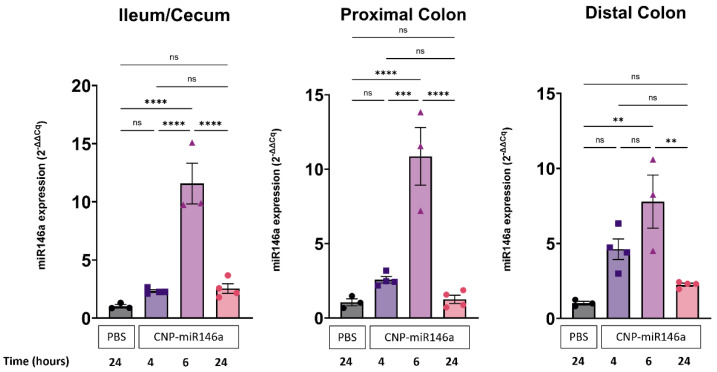
Transient delivery of exogenous miR146a in CNP to the gut mucosa. Oral administration of 5 ng of CNP-miR146a in 200 µL of PBS led to a significant increase in miR146a in the terminal ileum/cecum (*p* < 0.001), proximal colon (*p* < −0.001), and distal colon (*p* = 0.002) of naïve WT mice 6 h after gavage when compared to mice receiving 200 µL PBS alone. Asterisks indicate statistical significance (one-way ANOVA followed by Tukey multiple comparison test; ** *p* < 0.01, *** *p* < 0.005, **** *p* < 0.001, ns—not significant) and bars represent means with standard error of mean (SEM).

Endogenous miR146a and CNP-miR146a cannot be distinguished in qRT-PCR analysis. Therefore, expression of nanoparticle-derived miR146a can only be observed on the background of endogenous murine miR146a levels. Compared to mice that received 200 µL PBS alone (measure of endogenous miR146a), mice that received oral gavage of 5 ng of CNP-miR146a demonstrated a significant increase in miR146a expression in the terminal ileum/cecum, as well as in the proximal and distal colon, at six hours post-administration (*p* < 0.001, *p* < 0.001, and *p* = 0.002, respectively) (Figure 2). Between 6 h and 24 h post-administration, mucosal miR146a expression decreased to levels indistinguishable from baseline. This suggests a relatively short life of the nanoparticle-derived miR146a in the gut mucosa and the need for daily administration in future clinical trials.

### 4.3. Oral Delivery of CNP-miR146a in Mice with Established Colitis Led to Significantly Reduced Inflammation and Leukocyte Infiltration Within Five Days of Treatment

**Figure 3:**
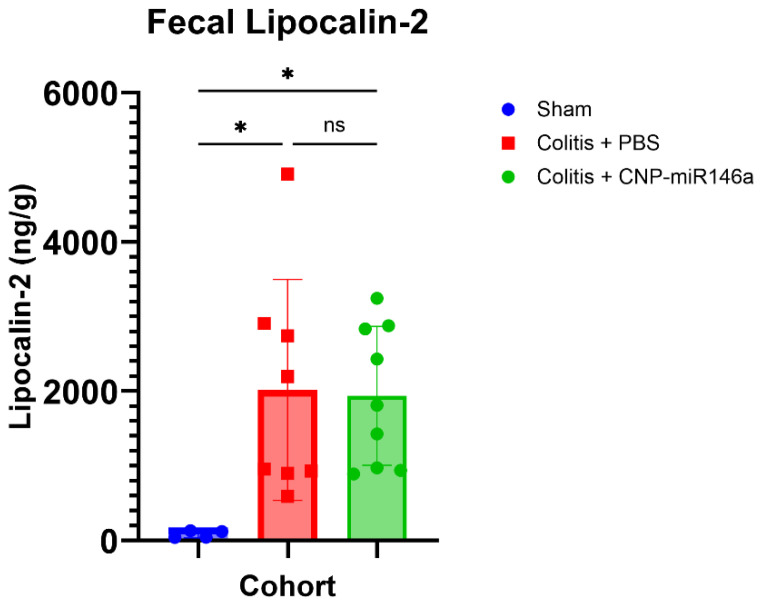
Fecal lipocalin-2 distribution in Rag2^−/−^ cohort mice. Rag2^−/−^ mice that received T cell transfers demonstrating fecal lipocalin-2 levels ≥ 900 ng/g 6–10 weeks later were evenly distributed to the PBS and CNP-miR146a treatment arms. There was no significant difference in average fecal lipocalin-2 levels between the two treatment cohorts. Rag2^−/−^ mice that received PBS injections and oral PBS did not demonstrate elevated fecal lipocalin-2 levels. Mean, SEM, and individual data points are indicated, and asterisks indicate statistical significance (one-way ANOVA followed by Tukey’s multiple comparison test; * *p* < 0.05, ns—not significant).

Rag2^−/−^ mice adoptively transferred with naïve T cells received daily oral gavage of PBS or CNP-miR146a for five consecutive days after they reached a predefined level of fecal lipocalin 2 (≥900 ng/g of fecal matter) as an indicator of established chronic colitis. Mice were evenly distributed to PBS or CNP-miR146a cohorts with no significant difference in fecal lipocalin 2 levels between the two groups (Figure 3).

**Figure 4:**
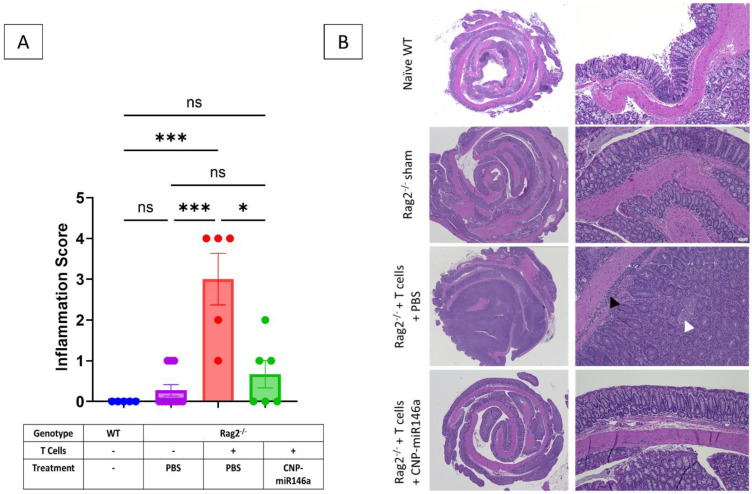
Oral CNP-miR146a administration significantly improves histological inflammation in the colon of mice treated with CNP-miR146a. (**A**) Representative H&E images of colonic histology on brightfield at 20× (**left**) or 100× (**right**) magnification. (**B**). Summary of quantitative unbiased analysis of colonic inflammation using scoring criteria depicted in Table 1. Data were analyzed with Kruskal–Wallis test. Mean, SEM, and individual data points are indicated. Asterisks indicate a statistical difference between groups (* *p* = 0.025, *** *p* < 0.001, ns—not significant).

Mice in both wild-type and sham control groups had colons with minimal to no inflammation. Colons of PBS-treated Rag2^−/−^ colitic mice demonstrated marked mucosal hyperplasia and inflammatory cell infiltration of the submucosa along with crypt abscesses, goblet cell loss, and erosions. Colons of CNP-miR146a-treated Rag2^−/−^ colitis mice showed minimal mucosal hyperplasia and reduced inflammatory cell infiltration mostly restricted to the mucosal layer (Figure 4A). Scoring of colonic inflammation reflected the observed histology and demonstrated increased inflammation in the colons of PBS-treated adoptively transferred Rag2^−/−^ mice as compared to WT naive controls and Rag2^−/−^ sham controls (*p* < 0.001 and *p* < 0.001, respectively). Rag2^−/−^ colitis mice treated with CNP-miR146a showed reduced inflammation scores in their colons as compared to PBS-treated Rag2^−/−^ colitis mice (*p* = 0.025), which became statistically indistinguishable from controls (Figure 4B).

**Figure 5:**
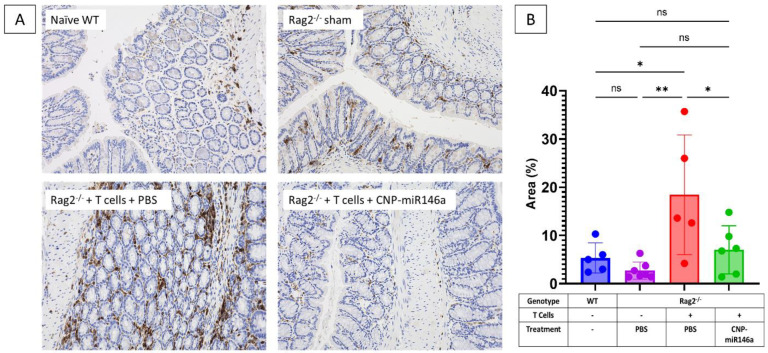
Oral CNP-miR146a administration reduces mucosal leukocyte infiltration in colitic mice. (**A**) Representative images of CD45^+^ immunohistochemical staining of the colons at 200× magnification. (**B**) Summary of quantitative analysis of CD45^+^ cell infiltration. Mean, SEM, and individual data points are indicated. Data were analyzed with one-way ANOVA with Tukey’s multiple comparisons test. Asterisks indicate statistical differences between groups (* *p* < 0.05, ** *p* = 0.003, ns—not significant).

Adoptive T cell colitis in PBS-treated mice was associated with increased mucosal infiltration by CD45^+^ leukocytes as compared to naïve WT and Rag2^−/−^ sham controls (*p* = 0.023 and *p* = 0.003, respectively). Oral gavage with CNP-miR146a resulted in a significant decrease in leukocyte infiltration as compared to PBS-treated colitic mice (*p* = 0.042) and reduced it to levels comparable to naïve WT or Rag2^−/−^ sham controls (Figure 5).

### 4.4. Oral CNP-miR146a Attenuates Oxidative Stress in the Inflamed Colon

**Figure 6:**
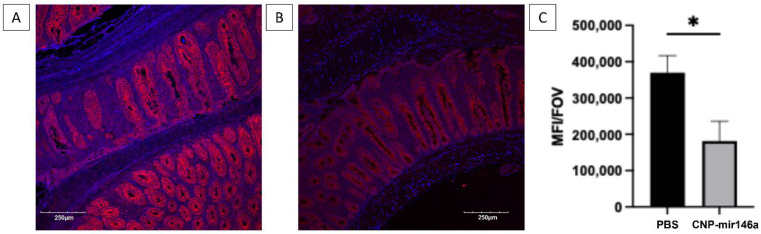
Oral CNP-miR146a administration attenuates oxidative stress in adoptive T cell transfer colitis. Representative 8-OHdG immunofluorescence images of the colons of PBS- (**A**) or CNP-miR146a-treated (**B**) mice with active colitis. (**C**) Summary of the quantitative analysis of the 8-OHdG-associated fluorescence (MFI/FOV—mean fluorescent intensity per field of view). Means and standard deviations are shown. Data were analyzed with Student’s *t*-test (n = 3 in each group; * *p* < 0.05).

CNPs are capable of protecting cells from oxidative damage due to their ability to efficiently scavenge cellular reactive oxygen species [42]. To test whether reduced oxidative stress may have contributed to the protective effects of CNP-miR146a, we used immunofluorescent detection of tissue 8-hydroxy-2′-deoxyguanosine (8-OHdG) as an established marker of oxidative DNA damage [43]. Colitic Rag2^−/−^ mice treated with CNP-miR146a demonstrated reduced intensity of 8-OHdG staining in their colons compared to Rag2^−/−^ colitic mice treated with PBS, as demonstrated qualitatively and quantitatively in Figure 6 (*p* = 0.011).

### 4.5. Oral Delivery of CNP-miR146a Reduces Colonic Mucosal Expression of IL-6 and TNF in Established Colitis

**Figure 7:**
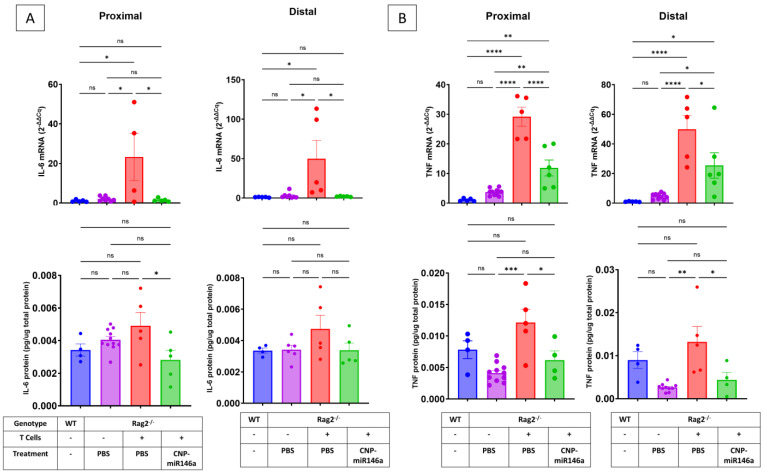
Oral CNP-miR146a administration attenuates colonic expression of IL-6 and TNF. qRT-PCR and ELISA were used to evaluate tissue expression of IL-6 (**A**) and TNF (**B**) transcripts and proteins, respectively. mRNA levels were analyzed by the comparative cycle threshold (Ct) method as a means of relative quantitation of gene expression, normalized to an endogenous reference (GAPDH), relative to a calibrator (normalized Ct value obtained from naïve WT mice), and expressed as 2^−ΔΔCt^ (Applied Biosystems User Bulletin number 2: Rev B “Relative Quantitation of Gene Expression”). IL-6 and TNF protein concentration in tissue was normalized per mg of total protein. Bars indicate means and SEM, with individual data point indicated. Asterisks indicate statistical significance on one-way ANOVA with Tukey’s multiple comparisons test (* *p* < 0.05, ** *p* < 0.01, *** *p* < 0.005, **** *p* < 0.001, ns—not significant).

miR146a deficiency is associated with increased responsiveness of macrophages to LPS and production of IL-6 and TNF [44], which both significantly contribute to IBD pathogenesis. Adoptive T cell colitis in PBS-treated mice predictably led to significantly increased expression of IL-6 and TNF mRNA in the colonic mucosa as compared to naïve WT mice or Rag2^−/−^ sham controls. Oral treatment with CNP-miR146a reduced expression of both cytokine transcripts, and in the case of IL-6 mRNA, to levels indistinguishable from healthy controls (Figure 4A,B). Protein quantification using ELISA demonstrated a significant reduction in IL-6 levels in the proximal colons of colitis Rag2^−/−^ mice treated with CNP-miR146a compared to PBS-treated colitis Rag2^−/−^ mice treated with PBS, with a similar, though not statistically significant trend, in the distal colon (Figure 7A). TNF protein levels were increased in the proximal and distal colons of colitic PBS-treated Rag2^−/−^ mice compared to sham Rag2^−/−^ controls and significantly reduced in colitic Rag2^−/−^ mice treated with CNP-miR146a (Figure 4B). There was no significant difference in colonic IL-6 or TNF protein expression between CNP-miR146a-treated colitic mice and Rag2^−/−^ sham controls (Figure 7A,B).

### 4.6. Oral Delivery of CNP-miR146a Reduced Mucosal Matrix Metalloproteinase 9 (MMP-9) Expression in Established Colitis

**Figure 8:**
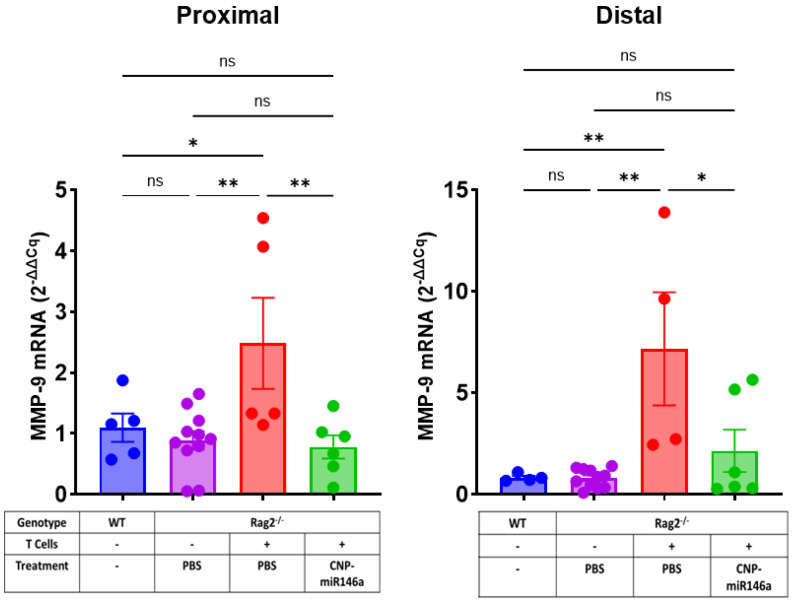
Oral CNP-miR146a administration attenuates colonic expression of MMP-9 mRNA. qRT-PCR was used to evaluate tissue expression of MMP-9 mRNA levels, which were analyzed by the comparative cycle threshold (Ct) method as described in Figure 6’s legend. Bars indicate means and SEM, with individual data point indicated. Asterisks indicate statistical significance on one-way ANOVA with Tukey’s multiple comparisons test (* *p* < 0.05, ** *p* < 0.01, ns—not significant).

MMP-9 was identified as an important marker of inflammation in both UC and CD [45,46], and targeting MMP-9 in preclinical models has led to attenuated colitis [47,48,49]. Although selective targeting of MMP-9 with monoclonal antibody in UC or CD patients did not induce a significant symptomatic or endoscopic response [50,51], a result that may be attributable to the duality of MMP-9 function depending on the biological process or stage of inflammation [52], MMP-9 was reported to be a target of miR146a in human breast cancer [53]. To test whether oral CNP-miR146a could affect colonic expression of MMP-9 during active inflammation, we analyzed its expression in the proximal and distal segments by qRT-PCR. Colitic Rag2^−/−^ mice treated with oral PBS showed elevated expression of mucosal MMP-9 compared to wild-type and sham controls in both proximal (*p* = 0.015 and *p* = 0.002, respectively) and distal (*p* = 0.008 and *p* = 0.001, respectively) colons. Rag2^−/−^ colitic mice treated with oral CNP-miR146a showed significantly reduced MMP-9 expression compared to PBS-treated mice in both segments (*p* = 0.003 and *p* = 0.023, respectively) and no significant difference compared to wild-type and sham controls (Figure 8).

### 4.7. Oral Delivery of CNP-miR146a Reduces Colonic Infiltration and/or Activation of Pathogenic Th1 and Th17 Cells

**Figure 9:**
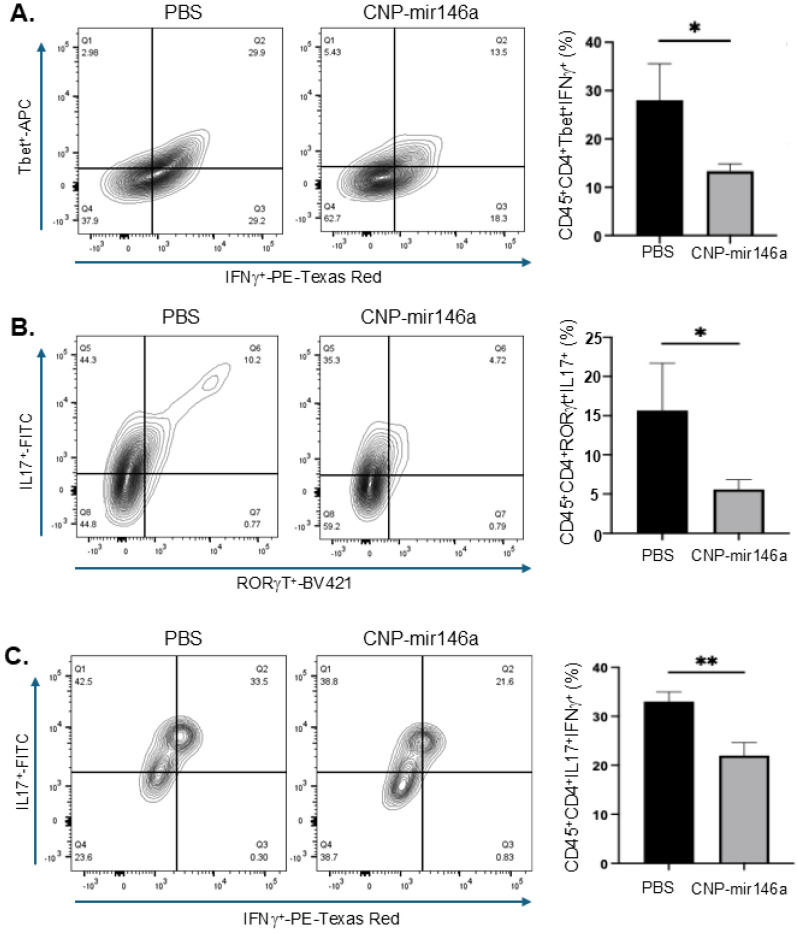
Oral CNP-miR146a restricts the expansion of pathogenic Th1 and Th17 cells in the colonic lamina propria. Representative contour plots and summary of flow cytometry analysis of (**A**) CD4^+^Tbet^+^IFNg^+^ Th1 cells, (**B**) CD4^+^RorgT^+^IL17^+^ Th17 cells, and (**C**) CD4^+^IFNg^+^IL17^+^ T cells. Bar graphs indicate means and standard deviation (SD). Data were analyzed with two-tailed *t*-test (n = 3, * *p* < 0.05, ** *p* < 0.01).

Oral treatment with CNP-miR146a in adoptively transferred colitis Rag2^−/−^ mice significantly reduced the expansion of colonic lamina propria (cLP) Th1 cell population, defined as CD4^+^Tbet^+^IFNg^+^ cells from approximately 30% in PBS-treated mice to approximately 13% in CNP-miR146a-treated mice (*p* < 0.05; Figure 6A). RORγt is the pivotal transcription factor that directs the differentiation of IL-17-producing inflammatory T cells. Similarly to the Th1 lineage, we observed a significant reduction CD4^+^IL-17^+^RORgT^+^ Th17 cells in colitic mice treated with CNP-miR146a nanoparticles (reduced from approximately 10% to 4%; *p* < 0.05; Figure 9A). Furthermore, our analysis of dual-positive IL-17^+^IFNg^+^ T cell population, considered to be Th17 transformed into Th1 lymphocyte precursors with high pathogenic potential [54,55,56], also indicated a significant reduction of this population with oral CNP-miR146a therapy (*p* < 0.01; Figure 9C). Combined with other outcomes, the reduction in Th1 and Th-17 cell populations in this T-cell mediated model of colitis suggests a strong immunomodulatory effect of CNP-miR146a nanoparticles.

### 4.8. Colonic miR146a Returns to Baseline Expression After Five Days of Oral Treatment with CNP-miR146a

**Figure 10:**
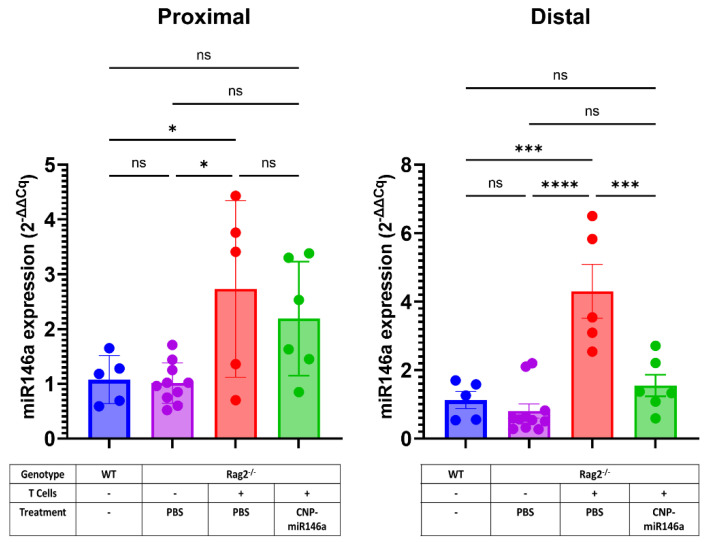
Colonic miR146a returns to baseline expression after five days of oral treatment with CNP-miR146a. Colonic expression of miR146a was evaluated in the proximal and distal colon 24 h after the last dose of CNP-miR146a using qRT-PCR and analyzed by the comparative cycle threshold (Ct) method as described in Figure 6’s legend. Bars represent means and SEM with individual data point indicated. Asterisks indicate statistical significance on one-way ANOVA with Tukey’s multiple comparisons test (* *p* < 0.05, *** *p* < 0.005, **** *p* < 0.001, ns—not significant).

Mucosal expression of miR146a is elevated in pediatric and adult UC and CD patients [57,58,59]. This likely represents an important yet insufficient homeostatic response to inflammation. Consistent with these observations, PBS-treated colitic Rag2^−/−^ mice had elevated expression of endogenous miR146a in the inflamed colonic mucosa. Seemingly paradoxically, CNP-miR146a-treated mice showed decreased expression of miR146a in the proximal (trend) and distal colon (*p* < 0.001; Figure 10). This analysis was conducted in tissues harvested 24 h after the last oral administration of CNP-miR146a and, considering the limited persistence of the exogenous miR146a (Figure 2), the observed miRNA expression at this time likely represented a combined but primarily endogenous miR146a. The observed decrease in mucosal miR146a in CNP-miR146a-treated mice should thus be interpreted as another biomarker of reduced inflammation.

## 5. Discussion

Attempts at oral delivery of RNA-based therapies have often been avoided given the challenges presented by the unstable nature of RNA coupled with the extreme environment of the gastrointestinal tract. To address these hazards, some have resorted to encapsulating gene-based therapies in protective materials like gelatin, synthetic polyesters, and even aminoglycoside antibiotics [60,61,62,63,64]. Although controversial, there are data suggesting that exogenous miRNAs can survive the mammalian gastrointestinal tract [65,66,67,68] and that the stability of miRNA depends on its individual structural properties and nucleic acid sequence [67]. Prior research has shown that orally delivered CNPs accumulate in the large intestines of both mice with colitis and healthy mice, with a proclivity towards diseased tissue [24]. Our study is the first to demonstrate the stability of CNP-miR146a in a simulated gastrointestinal tract, its oral availability, and impressive efficacy in a mouse model of established, chronic, progressive, T cell-mediated, and microbiome-dependent adoptive T cell transfer colitis. While no in vitro model perfectly simulates physiological conditions, the protocol used in this study, adapted from Minekus et al. [35], replicates many factors in the human gastrointestinal tract, including the concentrations of salts, acids, bases, and digestion time. Although the use of digestive proteases in this study was limited to trypsin, the most abundant of intestinal proteolytic enzymes, the chemical structure of CNP-miR146a makes it an unlikely target of proteolytic enzymes, as confirmed by its stability in simulated intestinal fluid. One study reported that a miR146a mimic was less stable compared to other miRNA in an in vitro digestion study [69]. We postulate that chemical conjugation of miR146a to CNP contributes to its stability in the gastrointestinal environment.

We also demonstrated an effective delivery of miR146a to the lower GI tract after oral gavage, with a peak at 6 h post-administration, consistent with the gastrointestinal transit time in mice [36]. Mucosal expression of exogenously delivered miR146a waned by 24 h, although our experimental design did not allow for higher time resolution of this effect. Importantly, the short lifespan of miR146a after oral delivery as CNP-miR146a should not be considered a pharmacokinetic flaw, since the dose and ease of oral administration, combined with the efficacy of quick elimination, may all indicate a safe and efficacious pharmacological approach to the treatment of IBD. The potential for CNP-miR146a to be orally delivered is significant considering its implications for clinical translation. Oral drugs have multiple advantages over other routes of administration. For example, oral drug delivery bypasses the need for intravenous access, which can be difficult to obtain in sick patients and in children, carries an increased risk of infection and thromboembolic events, and is more expensive. Additionally, oral drug use in IBD may have the unique advantage of increasing drug bioavailability to the intestinal tissues [60] while reducing systemic toxicity by requiring a hepatic first-pass metabolism.

In our study, CNP-miR146a was remarkably efficacious in reducing an established T cell-mediated experimental colitis in a widely accepted animal model of IBD which mimics Crohn’s disease pathophysiology [70,71] and was shown to exhibit alterations in gene expression that were similar to those in human Crohn’s disease [72]. Impressively, a daily dose of 5 ng of CNP-miR146a administered over five days was sufficient to reduce colonic inflammation to near baseline. This anti-inflammatory effect was reflected in improved colonic histology, reduced tissue oxidative stress, attenuation of mucosal proinflammatory cytokine expression, reduced immune cell infiltration, and reduced populations of pathogenic Th1 and Th17 cells, including double positive IL17^+^IFNg^+^ T cells.

The mechanism behind the therapeutic effect of CNP-miR146a in this model is likely through the combined action of miR146a on its targets and the antioxidative effects of CNP. Physiologically, miR146a expression is induced alongside activation of the NF-kB pathway by microbial TLR ligands and damage-associated molecular patterns (DAMPs) from injured cells [73,74]. miR146a is considered to be a part of a negative feedback loop to restrict the inflammatory response and facilitate the resolution phase. Among its effects, miR146a targets IRAK-1 and TRAF-6 to reduce their expression and prevents translocation of NF-kB to the nucleus by degrading these upstream mediators [12]. In IBD, the NF-kB pathway contributes to the aberrant TNF and IL-6 secretion by macrophages and T-lymphocytes that infiltrate the inflamed regions of the gut [9,75]. Given that miR146a expression is increased in IBD patients [8,74] and in our model of colitis, it is likely that the physiological rise in miR146a in response to NF-kB activation is insufficient in counteracting the overactivation of multiple inflammatory signaling pathways. Furthermore, certain polymorphisms in the miR146a gene have been associated with increased or decreased risk and/or severity of IBD in patients [13,76,77], which further strengthens its importance in the disease pathogenesis and highlights its potential as a therapeutic agent.

The second mechanism by which CNP-miR146a may work is via scavenging of ROS, which are a known contributor to dysregulated inflammation in IBD [78]. ROS are byproducts of inflammation, and persistent inflammatory states can produce excess ROS that overwhelm the cellular antioxidant defense mechanisms and lead to oxidative stress and subsequent cellular and molecular damage [79]. The colonic mucosa of Crohn’s disease patients has been shown to have excess ROS production and reduced components of antioxidant defense mechanisms [80,81]. In this study, we demonstrated that oral CNP-miR146a administration reduced oxidative DNA damage and the mucosal abundance of 8-OHdG, an established marker of oxidative DNA damage [43]. We have previously demonstrated that our CNP nanoparticles alone can reduce inflammation in other animal models, although this effect was less pronounced than when they were administered conjugated to miR146a [82,83].

The employed model of IBD, with all its advantages, also has drawbacks. It is not a reliable model of Crohn’s ileitis, and approximately a third of Crohn’s patients have isolated ileal disease. We could have also missed the effects of CNP-miR146a on B cells (absent in Rag2^−/−^ mice) or regulatory T cells (Tregs). The latter are not a part of the adoptively transferred naïve T cell pool, which differentiates into a very scarce Treg population in the gut of recipient mice. Since miR146a has been shown to contribute to Treg differentiation and is essential to the ability of Tregs to restrain pathogenic Th1 responses [84], relative lack of Tregs in the model of adoptive T cell transfer colitis may in fact lead to underestimation of the effects of CNP-miR146a by eliminating this additional immunosuppressive effect of miR146a. Furthermore, our study does not provide a translational model for preclinical evaluation of drugs targeted for ulcerative colitis. Moreover, at this point, we are not able to evaluate or discriminate the primary and secondary target cells of this therapeutic approach. Future work with fluorescently labeled CNP-miR146a will allow us to overcome this limitation.

## 6. Conclusions

The data from this study clearly demonstrate the therapeutic potential of orally administered CNP-miR146a for the treatment of Crohn’s colitis.

## Figures and Tables

**Table 1:** Stock simulated gastric fluid (SGF) and simulated intestinal fluid (SIF) were created in RNAse-free water with the addition of the listed salts to the designated concentrations.

	Simulated Gastric Fluid (SGF)	Simulated Intestinal Fluid (SIF)
Constituent	mM	mM
KCl	6.9	0.68
KH_2_PO_4_	0.9	0.08
NaHCO_3_	25	8.50
NaCl	47.2	3.84
MgCl (H_2_O_2_)	0.1	0.03

**Table 2:** Scoring system used for evaluation of colon histology. Reprinted/adapted with permission from Ref. [41]. Copyright 2014, IJCEP: https://creativecommons.org/licenses/by/4.0/.

Inflammatory Cell Infiltrate		Epithelial Changes	Mucosal Architecture	Score
Severity	Extent			
Minimal	Mucosa	Minimal hyperplasia		1
Mild	Mucosa, sometimes extending into submucosa	Mild hyperplasia, minimal goblet cell loss ± erosions		2
Moderate	Mucosa and submucosa	Moderate hyperplasia ± few crypt abscesses, moderate goblet cell loss ± erosions		3
Marked	Mucosa and submucosa	Marked hyperplasia ± several crypt abscesses and/or erosions	±Irregular crypts or crypt loss ± ulcerations	4
Marked	Transmural	Marked hyperplasia ± multiple crypt abscesses	±Irregular crypts or crypt loss ± ulcerations	5

## Data Availability

The raw data supporting the conclusions of this article will be made available by the authors upon request.

## Abbreviations

microRNA-146a (miR16a), cerium oxide nanoparticles (CNP), recombinant activating gene 2 knockout (Rag2^−/−^), 8-hydroxy-2′-deoxyguanosine (8-OHdG), inflammatory bowel disease (IBD), tumor necrosis factor (TNF), interleukin-23 (IL-23), Janus kinase (JAK), microRNAs (miRNAs), long non-coding RNAs (lncRNAs), nuclear factor kappa-light-chain-enhancer of activated B cells (NF-κB), interleukin-1 receptor-associated kinase 1 (IRAK1), tumor necrosis factor receptor-associated factor 6 (TRAF6), toll-like receptors (TLRs), reactive oxidative species (ROS), 2,4,6-Trinitrobenzenesulfonic acid (TNBS), 1′-1′ carbonyldiimidazole (CDI), inductively coupled plasma mass spectrometry (ICP-MS), phosphate buffer saline (PBS), simulated gastric fluid (SGF), simulated intestinal fluid (SIF), quantitative real-time polymerase chain reaction (qRT-PCR), intraperitoneal (IP), wild type (WT), ethylenediaminetetraacetic acid (EDTA), mean fluorescent intensity (MFI), interleukin-6 (IL-6), matrix metalloproteinase 9 (MMP-9), glyceraldehyde 3-phosphate dehydrogenase (GAPDH), standard error of mean (SEM), Hank’s balanced salt solution (HBSS), damage-associated molecular patterns (DAMPs).
